# Postoperative Management of Shivering: A Comparison of Pethidine vs. Ketamine

**DOI:** 10.5812/aapm.15499

**Published:** 2014-03-14

**Authors:** Mahmood Eydi, Samad EJ Golzari, Davood Aghamohammadi, Khosro Kolahdouzan, Saeid Safari, Zohreh Ostadi

**Affiliations:** 1 Department of Anesthesiology, Tabriz University of Medical Sciences, Tabriz, Iran; 2 Liver and Gastrointestinal Disease Research Center, Tabriz University of Medical Sciences, Tabriz, Iran; 3 Faculty of Paramedical, Tabriz University of Medical Sciences, Tabriz, Iran; 4 Department of Anesthesiology, Rasoul Akram Medical Center, Iran University of Medical Sciences (IUMS), Tehran, Iran

**Keywords:** Shivering, Ketamine, Meperidine, Anesthesia, Pethidine

## Abstract

**Background:**

One of the unpleasant side effects of general anesthesia is shivering in the process of recovery. It is an involuntary oscillatory mechanical movement that can be classified as clonic movements. These movements can affect one or several groups of skeletal muscles beginning from 5 to 30 minutes after the discontinuation of anesthesia.

**Objectives:**

We aimed to study ketamine’s effect on shivering after operation compared to pethidine as a way for treatment of postoperative shivering.

**Patients and Methods:**

In this study, 60 patients who underwent ENT surgery with general anesthesia and had shivering during recovery were randomly divided into two groups of 30 patients each receiving ketamine (0.2 mg/kg IV) and pethidine (0.5 mg/kg).

**Results:**

There was no statistically significant difference between the shivering intensity in both groups. Only regarding the shivering in the first minute after entering the recovery room, there was an obvious difference between ketamine and pethidine groups which was again not statistically significant (P = 0.07).

**Conclusions:**

The results of this study showed that ketamine and pethidine are both equally effective in the reduction of postoperative shivering.

## 1. Background

Management of patients’ complaints has always been of priority for the physicians (1-3). Post-operative complications are major causes of the morbidity in surgical patients (4-6). Shivering, asynchronous and random spontaneous contractions of skeletal muscles to increase basal metabolism, is an important defense mechanism for temperature regulation in adults (7). Shivering is one of the unpleasant complications during recovery from anesthesia manifesting as involuntary oscillatory mechanical movements and can be characterized as clonic movements starting from 5 to 30 minutes after the cessation of anesthesia (7). Although it is unclear what percentage of the patients experience shivering, its occurrence has been reported to vary from 5% to 66% in patients recovering from anesthesia. Post-operative shivering in patients severely increases oxygen consumption (400 times) which is followed by a rise in CO_2_ production leading to acidosis when the alveolar ventilation is not increased proportionally (7). Not only anesthetic drugs impair normal body temperature regulation but also patients under general anesthesia are exposed to cold temperature. Heat intemperance is increased by vasoconstriction inhibition, decreased metabolic rate and shivering is inhibited by anesthetic agents and muscle relaxants. Although its mechanism of action is not completely understood, pethidine probably acts directly on the thermoregulatory centre or via opioid receptors. It is likely that N-methyl-d-aspartate (NMDA) receptor antagonists also modulate thermoregulation at multiple levels. Like morphine, pethidine exerts its analgesic effects by acting as an agonist at the μ-opioid receptor (8). It also has an agonistic κ-opioid receptor action, which may be involved in the anti-shivering effects it elicits (9, 10).

Ketamine, derived from phencyclidine, is an agent used in dissociative anesthesia and characterized by thalamus and limbic system separation on the electroencephalograph (EEG). Fast acting and fat solubility of ketamine guarantee its rapid onset. Similar to single intravenous dose of other intravenous anesthetics, ketamine’s effect ends by its diffusion to passive tissues. The major metabolite of ketamine, norketamine is less powerful than ketamine and is excreted to the urine (8). Ketamine has characteristics such as induction of amnesia, cerebral vasodilatation, and ICP increase, marked and transient increase in blood pressure by stimulating sympathetic system, bronchial smooth muscle relaxation, analgesia and hallucination. Ketamine, which is a competitive NMDA receptor antagonist, has been shown to inhibit postoperative shivering in some reports and studies (11-13). Ketamine induction dose is 1-2 mg/kg as IV or 4-6 mg/kg IM. Low doses of Ketamine (0.2 to 0.8) are used for analgesia by some practioners. Ketamine provides effective analgesia without compromising the airway (7). Side effects of ketamine are mostly seen in high doses. Side effects have not been reported when ketamine was used at 0.5mg/kg about 20 minutes before the end of surgery or general anesthesia.

## 2. Objectives

We aimed to study ketamine’s effect on shivering after operation compared to Pethidine as a way for treatment of postoperative shivering.

## 3. Patients and Methods

After obtaining approval of the Ethical Committee of Tabriz University of Medical Sciences and informed written consent from patients, 60 subjects with ASA class or II undergoing rhinoplasty under general anesthesia who experienced post-operative shivering were selected randomly and divided into two groups (each group 30 patient), one group receiving ketamine (0.2 mg/kg IV) and other pethidine (0.5 mg/kg). To determine the sample size, online software was used (please see: http://www.stat.ubc.ca/~rollin/stats/size/). Simple randomization method was used in order to randomize the patients into two groups. Inclusion criteria were as follows: ENT surgeries under general anesthesia, patients with ASA class I or II, operation duration of 1-2 hours, occurrence of peri-operative shivering. Exclusion criteria were: patients with ASA class III or higher, emergency operations and patients with a history of shivering or thermal disorders. 

For induction of anesthesia, midazolam (2 mg), fentanyl (1-2 µg/kg) and propofol (3 mg/kg) were used in all patients and atropine (0.5 mg) and neostigmine (1.5 mg) were used at the end of operation for neuromuscular blockade antagonization. Duration of surgery, amount of infused fluid and blood loss throughout the procedures were noted. The axillary temperature was measured for all patients. Operation room temperature was maintained at 22 ^o^C. Patients’ shivering was graded in the recovery room and 60 patients were studied with postoperative shivering. Shivering of patients was grouped into five grades; grade 0: lack of shivering, grade I: slight shivering (inconsiderable yet apparent peripheral vasoconstriction), grade II: medium level shivering (muscular activity in one muscle group only), grade III: severe shivering (muscular activity in more than one muscle group without generalized shivering), grade IV: generalized shivering.

All patients in the recovery room received a blanket and oxygen by facial mask (4 L/min). Patients with shivering in the recovery room were randomly divided into two groups of 30 patients and in one group pethidine (0.5 mg/kg) and in the second group Ketamine (0.2 mg/kg) were used. Due to the high incidence of postoperative shivering no control group was utilized. Drugs diluted into a volume of 2 mL in coded syringes were injected by anesthesiologists that did not know either patients' management details or their shivering grade. Shivering grade was evaluated before (0 min) and every minute until 10 minutes after the treatment. Arterial oxygen saturation, heart rate and non-invasive blood pressure (systolic, diastolic and mean arterial blood pressure) of patients were recorded before and after treatment every minute for 10 minutes. Patients' temperature was measured immediately before treatment (zero minute) and 5 and 10 minutes after the treatment. Possible side effects associated with drug like nausea, vomiting, hypotension, hypertension, tachycardia, nystagmus, sense of walking in the air and hallucination was recorded. All variables were analyzed by SPSS 16 software. Descriptive statistical methods (frequency, percentage and mean ± SD) were used for the statistical evaluation. Chi square test was used for the comparison of qualitative findings. P value less than 0.05 was considered significant in the study. 

## 4. Results

 Patients in both groups were in 15 to 55 years old age range. There were no statistically significant differences regarding the gender, weight and physical condition (ASA) between the two groups (Table 1).

The measured axillary temperature for the patients in Groups K and P were 36.85 ± 0.28 ^o^C and 37.05 ± 0.27 ^o^C, respectively. The mean liquid volume administered throughout the procedures for the patients in Groups K and P were 1500 ± 500 mL and 1440 ± 400 mL, respectively. Mean systolic blood pressure of patients in the ketamine group was 123 ± 28 mmHg and mean diastolic blood pressure in these patients was 78.6 ± 13.56 mm Hg. Average pulse rate in patients was 97 ± 13 beats per minute, the average respiratory rate was 15.5 ± 1.5 breaths per minute, the average body temperature of patients was 36.85 ± 0.28 °C, the mean arterial oxygen saturation (Sao_2_) in patients was 97.75 ± 1.18%. Mean anesthesia duration in the ketamine group was 128 ± 43 min (Table 2). All these 30 patients received 1-2 μg/kg fentanyl. For the management of anesthesia in this group patients were anesthetized by propofol with a relaxant (cis-atracurium).

In this group of patients, nine patients (30%) had nausea, eight patients (26.7%) had tachycardia, two patients (6.7%) had nausea and tachycardia and one patient had vomiting while 10 patients (33.3%) had no side effects except shivering (Table 3). The fluid therapy throughout the operation: 80% of patients received Ringer and only 20% received normal saline. Mean volume of the received fluid was 1.5 ± 0.5 liter and minimum and maximum injected fluid volumes were 1 and 2.5 liters, respectively.

Sedation rate of all patients was evaluated moments after entering the recovery room (time zero), 10 minutes, 30 minutes and 45 minutes with 5 grades from zero to 4; alert status was recorded as Grade 0, waking up to sound as Grade 1, waking up with verbal command as grade 2, waking up with touching stimulation as grade 3, no response to sever stimulation as grade 4. Consequently, in the ketamine group, 9 patients (30%) were in Grade 2, 7 patients (23.3%) were in grade 4 and 14 patients (46.7%) were in Grade 3 at the time of entering recovery. Level of consciousness in patients was determined 10 minutes after the arrival of the patient in the recovery room, at this time 13 patients (43.3%) were in grade 3, 15 patients (50%) in grade 2, and 2 patients (6.7%) in grade 1. Sedation levels 30 minutes after arrival in the recovery room were categorized as below: 21 patients (70%) in Grade 1, 6 patients (20%) in Grade 2 and eventually 3 patients (10%) in grade 0. All patients were fully alert after 45 minutes and were classified as grade zero.

Mean systolic blood pressure in the pethidine group was 123 ± 28 mmHg and mean diastolic blood pressure was 74.06 ± 12.81 mmHg. Mean pulse rate in patients was 96 ± 12 beats per minute, mean RR 15.5 ± 1.5 breaths per minute and mean temperature 37.05 ± 0.27 °C, respectively. Mean arterial oxygen saturation (Sao_2_) was 92.06 ± 1.92%. Mean duration of anesthesia in the pethidine group was 144 ± 41 min (Table 2). All these 30 patients had received 1-2 μg/kg fentanyls preoperatively like the ketamine group and for all patients the same type of anesthetic drug (propofol) and relaxant (cis-atracurium) were administered for anesthesia maintenance. In the pethidine group, 12 patients (40%) had nausea, 6 patients (20%) tachycardia, 5 patients (16.7%) nausea and tachycardia and 1 patient (3.3%) vomiting; 6 patients did not have any complication except shivering (Table 3). Eighty four percent of patients received ringer and only 16% of them got normal saline. Mean fluid intake during operation was 1.44 ± 0.45 in the pethidine group.

**Table 1.** Demographic Characteristics and Physical Condition of Patients in the Ketamine and Pethidine Groups ^a, b^

Variable	Ketamine Group	Pethidine Group	P Value
**Age, y**	29.33 ± 12	30.5 ± 10.5	0.27
**Sex**			
Woman	15 (50)	15 (50)	-
Man	15 (50)	15 (50)	-
**Weight, kg**	10 ± 65	68 ± 7.5	0.24
**ASA**			
Class I	27	26	0.74
Class II	3	4	0.66

^a^ Abbreviation: ASA, American Society of Anesthesiologists.

^b^ Data are presented as mean ± SD or No. (%).

**Table 2.** Mean and Standard Deviation of Systolic and Diastolic Blood Pressure, Pulse Rate, Respiratory Rate, Temperature, Arterial Oxygen Saturation, and Duration of Anesthesia in Pethidine and Ketamine Groups

	Mean ± SD	Mean ± SD	P Value
**Systolic blood pressure, mmHg**	28 ± 123	28 ± 123	0.86
**Diastolic blood pressure, mmHg**	78.6 ± 13.56	74.06 ± 12.81	0.74
**Pulse rate, beats per minute**	13 ± 97	12 ± 96	0.63
**Respiratory rate, breaths per minute**	15.5 ± 1.5	15.5 ± 1.5	0.34
**Body temperature, ° C**	36.85 ± 0.28	37.05 ± 0.27	0.12
**Arterial oxygen saturation**	97.75 ± 1.18	97.06 ± 1.92	0.65
**Duration of anesthesia, min**	43 ± 128	41 ± 144	0.58

**Table 3.** Nausea, Vomiting, Tachycardia, Nausea and Tachycardia and Tremor Rate in Patients upon Arrival in the Recovery Room ^a^

Complication	Ketamine Group	PethidineGroup
**Nausea**	9 (30)	12 (40)
**Vomiting**	1 (3.3)	1 (3.3)
**Tachycardia**	8 (26.7)	6 (20)
**Tachycardia and nausea**	2 (6.7)	5 (16.7)
**Only shivering**	10 (33.3)	6 (20)

^a^ Data are presented as No. (%).

Sedation rate of these patients after entering into the recovery room was 12 patients in grade 2 (40%), 13 patients in grade 3 (43.3%) and 5 patients grade 4 (16.7%). Level of consciousness in patients was determined 10 minutes after the arrival into the recovery and at this time 11 patients (36.7%) were in grade 2, 9 patients (30%) in grade 3, 8 patients (26.7%) in grade 1 and 2 patients in grade 4 (6.7%). Sedation levels 30 minutes after arrival to the recovery were categorized as below: 14 patients (46.7%) in Grade 1, 10 patients (33.3%) in Grade 0 and eventually 6 patients (20%) in grade 2. Seventy percent of patients were fully alert after 45 minutes and were classified as grade zero and 30% were in grade 1. Shivering of patients was evaluated and grouped in five grades (from 0 to 4) every minute until 10 minutes as below; Grade 0: lack of shivering, grade I: slight but apparent shivering, grade II: muscular activity in one muscle group only, grade III: muscular activity in more than one muscle group without generalized shivering, grade IV: generalized shivering.

In the ketamine group 15 patients had grade 4, 14 patient grade 3 and 1 patient grade 1 shivering during start of the shivering based on the mentioned criteria; whereas 21 patient had grade 4 and 9 patient grade 3 shivering in the pethidine group. These results were not statistically different (P = 0.21) (Table 4). In minute 5 after the start of shivering, 8 patients had grade 0, 7 patients grade 1 and 2 patients grade 2 shivering in the ketamine group; also pethidine group had 6 grade 0 patients, 7 grade 1 patients and 2 grade 2 patients but the data were not statistically different (P = 0.48) (Table 4). In minute 7 after the start of shivering, 2 patients had grade 1, 1 patient grade 2 and others were without any shivering; also the pethidine group had 3 grade 1 patients and other patients were without shivering but data were not statistically different (P = 0.48) (Table 4). Patients of both groups did not have shivering after minute 8.

**Table 4.** The Intensity of Shivering in Patients upon Arrival in the Recovery Room, Minutes 5 and 7 in Both Ketamine and Pethidine Groups

Shivering Severity	Ketamine Group	Pethidine Group	P Value
**On Enterance**			0.21
Grade 0	0	0	
Grade 1	0	0	
Grade 2	1	0	
Grade 3	14	9	
Grade 4	15	21	
**Min 5**			0.86
Grade 0	8	6	
Grade 1	10	7	
Grade 2	3	2	
Grade 3	1	0	
Grade 4	0	0	
**Min 7**			0.48
Grade 0	0	0	
Grade 1	2	3	
Grade 2	1	0	
Grade 3	0	0	
Grade 4	0	0	

## 5. Discussion

Postoperative shivering is a common problem emerging from the general anesthesia in the recovery room that occurs in 5 to 65 percent of patients. This condition is very inconvenient for patients. Postoperative shivering is a phenomenon regulated by temperature (a physiological response to anesthesia-induced central hypothermia) or triggered by cytokine release induced by surgical procedures (14). Shivering is defined as the involuntary movement of one or more muscle groups during the first stage after general or local anesthesia. Furthermore, shivering is associated with other complications including increased oxygen consumption, decreased tissue oxygenation, increased production of carbon dioxide, lactic acidosis, increased left ventricular systolic work index, increased intraocular pressure of eye and brain, interference with ECG monitoring and fluctuations in the blood pressure (14). Pethidine is the most effective treatment for postoperative shivering. Ketamine is an anesthetic agent and non-competitive antagonist of N-methyl D-aspartate (NMDA) which in lower subanesthetic doses has analgesic effects, regulates temperature in multiple stages and prevents shivering (15, 16). This study showed that the use of pethidine (5 mg/kg) as opposed to the ketamine in patients (0.2 mg/kg) has no significant beneficial effect on postoperative shivering.

Also another study reported no significant difference between ketamine and pethidine in controlling postoperative shivering; however, pethidine is the medication of choice due to the high incidence of side effects in the ketamine group (17, 18). In a similar study, no case of shivering was reported in the pethidine group. In addition, use of prophylactic low dose of ketamine in shivering control after anesthesia for tonsillectomy in children was more effective than pethidine (19). In another study it was found that 0.5 mg/kg ketamine was better than 0.3 mg ketamine; however, pethidine is still the first and best choice (20). Unlike the present study, the only study on the effect of ketamine on shivering control reported that shivering was controlled after 4 minutes (21). In our study, no significant difference could be found between ketamine and pethedine anti-shivering features.

Although most studies agree with the anti-shivering effect of ketamine, pethidine is reported to be a better and more effective choice for shivering control in most of studies. Our study demonstrated that both ketamine and pethidine possess anti-shivering effects yet no significant difference exists between these agents. Considering the fact that no significant difference could be observed between both medications regarding post-operative shivering and also the fact that administration of Ketamine could be associated with some undesirable complications, the conventional administration of mepridine seems to be more logical and safer.
